# Validation and use of microdialysis for determination of pharmacokinetic properties of the chemotherapeutic agent mitomycin C - an experimental study

**DOI:** 10.1186/1471-2407-10-469

**Published:** 2010-09-01

**Authors:** Olaf Sørensen, Anders Andersen, Harald Olsen, Kristian Alexandr, Per Olaf Ekstrøm, Karl-Erik Giercksky, Kjersti Flatmark

**Affiliations:** 1 Department of Surgical Oncology, The Norwegian Radium Hospital, Oslo University Hospital, Montebello, 0310 Oslo, Norway; 2 Department of Clinical Pharmacology, The Norwegian Radium Hospital, Oslo University Hospital, Montebello, 0310 Oslo, Norway; 3 Department of Tumor Biology, Institute for Cancer Research, The Norwegian Radium Hospital, Oslo University Hospital, Montebello, 0310 Oslo, Norway; 4 Norwegian Radium Hospital Faculty Division, University of Oslo, 0310 Oslo, Norway

## Abstract

**Background:**

Mitomycin C is a chemotherapeutic agent used in the treatment of peritoneal surface malignancies, administered as hyperthermic intraperitoneal chemotherapy after cytoreductive surgery. Pharmacokinetic studies have been based on analyses of blood, urine and abdominal perfusate, but actual tissue concentrations of the drug have never been determined. Microdialysis is an established method for continuous monitoring of low-molecular substances in tissues, and in the present study microdialysis of mitomycin C was studied in vitro and in vivo.

**Methods:**

Using in vitro microdialysis, relative recovery was determined when varying drug concentration, temperature and perfusion flow rate. In vivo microdialysis was performed in rats to verify long-term stability of relative recovery in four compartments (vein, peritoneum, extraperitoneal space and hind leg muscle). Subsequently, intravenous and intraperitoneal bolus infusion experiments were performed and pharmacokinetic parameters were calculated.

**Results:**

In vitro, compatibility of mitomycin C and microdialysis equipment was demonstrated, and relative recovery was stable over an adequate concentration range, moderately increased by raising medium temperature and increased when flow rate was reduced, all according to theory. In vivo, stable relative recovery was observed over seven hours. Mitomycin C exhibited fast and even distribution in rat tissues, and equal bioavailability was achieved by intravenous and intraperitoneal infusion. The half-life of mitomycin C calculated after intravenous infusion was 40 minutes.

**Conclusions:**

Mitomycin C concentration can be reliable monitored in vivo using microdialysis, suggesting that this technique can be used in pharmacokinetic studies of this drug during hyperthermic intraperitoneal chemotherapy.

## Background

The chemotherapeutic agent mitomycin C (MMC) has previously been used in treatment of a wide specter of malignancies, in particular gastrointestinal, lung, head and neck and gynecological cancer [1]. Although mostly replaced by drugs with higher response rate and less toxicity, it still plays a role in curative treatment of anal cancer in combination with 5-flurouracil and radiation therapy and is also used in palliative chemotherapy [2,3]. The wide clinical anti-cancer effect in combination with specific properties such as non-cell cycle specific and direct cytotoxic effect has also rendered MMC an attractive drug for use by topical administration, utilized in the treatment of as diverse malignancies as bladder cancer and certain tumors of the eye [4,5]. During the last two decades MMC has also gained importance in the treatment of peritoneal surface malignancies, in particular pseudomyxoma peritonei (PMP), a low-grade malignant disease characterized by growth of mucinous tumors on the peritoneal surface [5-7]. PMP is treated by a two step strategy; surgical cytoreduction aimed to remove all macroscopic tumor and subsequent administration of MMC by hyperthermic intraperitoneal chemotherapy (HIPEC) for elimination of residual microscopic disease and free tumor cells [8-10]. Although the efficacy of HIPEC has not been proven in randomized controlled trials, the use of this treatment strategy has in recent years been extended to include selected patients with peritoneal carcinomatosis from more aggressive malignancies, mainly colorectal cancer [11,12].

In peritoneal surface malignancies the peritoneum is considered to be a relative barrier against locally invasive growth and systemic tumor spread. As long as the disease is confined to the abdominal cavity, local administration of MMC in the form of HIPEC is an attractive principle, since high intraabdominal drug concentration is achieved relative to systemic absorption, which is the main dose-limiting factor. Pharmacologic studies of MMC during HIPEC have hitherto been based on concentration analysis of blood, urine and abdominal perfusate collected during the procedure, but actual tissue concentrations have never been measured [13-16].

Microdialysis (MD) is a technique which enables continuous monitoring of the free extracellular concentration of solutes in tissues [17,18]. In MD, small-caliber probes with a semipermeable membrane connected to inflow and outflow tubes are perfused at a low flow rate with a liquid of osmotic properties similar to extracellular fluid. Small molecules are passively exchanged across the membrane between the perfusate and probe surroundings along the concentration gradient, and dialysate is collected from the outflow tube and the concentration of the solute in question is determined. Because of the continuous flow through the MD probe, concentration equilibrium over the membrane will never occur. A key step in MD-experiments is therefore calibration of the probe to determine relative recovery (RR), the ratio between the concentration in dialysate and the free extracellular concentration of the solute [19,20]. RR is determined by numerous factors, of which the most important are the perfusion flow rate, the area and permeability of the probe membrane and physiologic properties of the medium surrounding the membrane [21]. Because of the joint complexity of the factors which determine RR, individual calibration of the probes is required in each experiment [21,22].

In the present work a set of in vitro experiments was carried out, verifying compatibility of relevant MD equipment and MMC and showing that RR was influenced according to theory when perfusion flow rate, temperature or solute concentration were varied. In vivo MD was then performed to assess whether stable RR could be achieved over an adequate period of time, and finally the tissue distribution of MMC in rats following intravenous (i.v.) and intraperitoneal (i.p.) infusions was studied, allowing subsequent analysis of important pharmacokinetic properties of MMC.

## Methods

### Solutions and Standards

MMC (Medac, Hamburg, Germany) was dissolved in Glucose 5% to a stock concentration of 3 mM (1 mg/ml), aliquoted and stored in liquid nitrogen. Calibrators at 100, 10, 1 and 0.1 μM were made up in Ringer's-Acetate (R-A) (Fresenius Kabi, Halden, Norway) and stored at -70°C for up to two years without notable degradation. Aliquots of these calibrators were thawed, diluted, and included in each analytical series for calibration and quality control. MMC in microdialysates was quantified against calibrators in R-A. The same calibrators were diluted in rat plasma at the day of use for quantification of MMC in plasma samples. The internal standard solution was 5 μM tinidazole (T3021, Sigma-Aldrich Company Ltd, St. Louis, MO, USA) dissolved in R-A. Ethyl acetate, hexane and HPLC grade methanol were from Merck (Darmstadt, Germany). All aqueous reagents were made up in water purified through a Milli-Q UF-PLUS system (MillPore Corp., Bedford, MA, USA).

### Determination of mitomycin C concentrations in plasma and microdialysates

MMC concentrations in plasma were determined by reversed phase high performance liquid chromatography (HPLC) after solid-phase extraction (SPE). The analytical method and routines for sample handling was based on previously published studies [23-25]. Some modifications to the methods were made, e.g. the internal standard used was no longer available and adaptation to differences in equipment and instruments used. Chromatographic analysis was performed on an Agilent 1100 system (Agilent Technologies, Santa Clara, California, USA) with a G1311A Quaternary pump, G1379A degasser, G1313A autosampler, G1316A Thermostated Column compartment and a G1365B Multi-Wavelength Detector. System control and data acquisition and integration were performed by Chemstation software Rev. B. 02.01. The mobile phase buffer was a 20 mM potassium phosphate buffer pH 7.0. The chromatography system was set up with buffer/methanol: 90/10 in reservoir A and buffer/methanol: 60/40 in reservoir B delivered in equal portions with a flow rate of 0.5 ml/min at 50°C. The column used for separation was a Supelcosil LC-18, 150 × 3 mm with 3 μm particles protected by a 20 × 2.1 mm guard column (Supelco, Bellefonte, PA, USA). Tinidazole was eluted at 2.8 minutes and MMC at 4.2 minutes, followed by a 4-minute washout with 100% B to eliminate late eluting peaks. One sample was injected every 15 minutes. When analyzing microdialysates no sample preparation was necessary. Ten μl of mobile phase A was added to 10 μl of dialysate and 15 μl of the mixture was injected. For these samples the washout with 100% B was not required, and samples could be injected every 5 minutes. For extraction of plasma samples, 1-ml Isolute SPE columns containing 25 mg of endcapped C18 sorbent were used (International Sorbent Technology LTD, Hengoed Mid Glamorgan, UK). To each 25-μl plasma sample, 150 μl of internal standard solutions and 25 μl R-A (or calibrator solution) were added. All plasma samples were split and extracted in duplicate by loading approximately half of the volume onto each of two SPE-columns. All steps up to the second wash (5% methanol) were performed without vacuum, the remaining steps were performed using gentle vacuum to pull the liquids through the columns. The columns were preconditioned with 1 ml methanol and 1 ml water. After loading, columns were washed with 0.5 ml R-A, 0.2 ml 5% methanol in water, and 0.2 ml hexane. Columns were dried by flushing with air for 2-3 minutes prior to elution using two times 100 μl of a 50/50 mixture of methanol and ethyl acetate. The eluates were evaporated to dryness for 30 minutes in a vacuum centrifuge. The residues were dissolved in 25 μl of mobile phase A, centrifuged and 15 μl of sample was injected onto the HPLC system.

The HPLC assays were run with the acceptance/rejection criteria suggested by Shah et al [26,27]. The lower limit of quantization for the determination of total plasma concentrations of MMC was 0.1 μM. The upper limit was 4 μM. Within this range the method had a median accuracy of 100% (range 92%-111%), with a precision of 0.1%-6%. The lower detection limit for the determination of MMC in microdialysates was 0.02 μM. The tested upper limit was 100 μM, but the assay was set up with 10 μM as upper limit for the in-vivo studies. The method had a median accuracy of 99% (range 97%-103%), with a precision from 2% at MMC concentration 100 μM to 13% at MMC concentration 0.02 μM.

### Microdialysis

MD was performed using CMA/20 Elite microdialysis probes with membrane length 10 mm, diameter 0.5 mm and molecular cutoff 20 kDa. A CMA/100 syringe pump, 1-ml glass micro syringes and a CMA/140 fraction collector, all from CMA/Microdialysis AB (Solna, Sweden) were used to administer perfusion solution and handle collected fractions. MD fractions were collected in glass vials, diluted 1:1 with mobile phase A and stored at -70°C until analysis.

### In vitro microdialysis in Ringer's - Acetate

The general mathematical expression of RR is:

(1) $$\text{RR}\left(\%\right)=\text{1}00\times \left({\text{C}}_{\text{in}}-{\text{C}}_{\text{out}}\right)/\left({\text{C}}_{\text{in}}-{\text{C}}_{\text{m}}\right)$$

in which C_in_, C_out _and C_m _is the concentration in perfusate, dialysate and the medium surrounding the probe membrane, respectively [28]. Two experimental setups were applied in order to compare in vitro RR by gain (RR_gain_) and RR by loss (RR_loss_). To determine RR_gain_, the probes were immersed in R-A spiked with MMC to concentrations 1, 5 and 10 μM and perfused with blank R-A. In this case C_in _= 0, and RR_gain _was calculated by the equation:

(2) $${\text{RR}}_{\text{gain}}\left(\%\right)=\text{1}00\times {\text{C}}_{\text{out}}/{\text{C}}_{\text{m}}$$

The same probes were used to determine RR_loss _by the retrodialysis method, in which identical concentrations of MMC in R-A were used as perfusate and the probes were immersed in blank R-A. In this case C_m _= 0, and RR_loss _was calculated by the equation:

(3) $${\text{RR}}_{\text{loss}}\left(\%\right)=\text{1}00\times \left({\text{C}}_{\text{in}}-{\text{C}}_{\text{out}}\right)/{\text{C}}_{\text{in}}$$

The experiments were conducted for four hours at 37°C with flow rate 1 μl/min and sample volume 20 μl, i.e. 12 replicates were collected from each probe.

### In vitro microdialysis in plasma

To assess the influence on RR by changing the concentration, perfusion flow rate and temperature, three sets of experiments were performed with MD probes immersed in male rat plasma spiked with MMC at concentrations 1.0, 2.5 and 5.0 μM. Unless otherwise stated, the temperature in the medium was 37°C and flow rate was 1 μl/min. All experiments were performed in triplicate and the mean of three 10-μl samples was calculated for each data point. The experiments with variable flow rate (flow rate experiments) were performed with flow 6, 4, 2, 1 and 0.5 μl/min, and the temperature experiments were performed at 37, 40, 42 and 44°C. In these experimental setups RR was calculated using equation (2), in which C_m _was the concentration of MMC in plasma. The zero-net flux (ZNF) method can be used both to calculate the solute concentration and for probe calibration, provided a stable concentration of the solute in the medium. In ZNF the perfusate contains the solute of interest and by varying C_in _in a stepwise manner and measuring the resulting C_out_, C_m _and RR can be calculated using equation (1) [29]. ΔC (=C_out_-C_in_) is plotted against C_in _and a linear regression line is drawn between these points. The point of interception of the regression line on the x-axis (ΔC = 0) is where C_in _is equal to the free fraction of C_m_, while the slope of the regression line represents RR for the probe [30]. The ZNF-experiments were performed with C_in _0.5, 4 and 10 μM.

### Animals

Fifteen locally bred Rowett nude male rats with mean weight 346 g (range 302-396) were used, divided into three groups. Four animals were used to assess the stability of RR by the retrodialysis method in four different compartments over seven hours. For pharmacokinetic studies, six animals were given i.v. and five were given i.p. infusions of MMC. The animals were maintained under specific pathogen-free conditions, and food and water were supplied ad libitum. Housing and all procedures involving animals were performed according to protocols approved by the animal care and use committee.

### Surgical procedures

Anesthesia was initialized with isoflurane (Sevorane, Abbott Scandinavia AB, Solna, Sweden) and maintained with subcutaneous (s.c.) injections of a mixture of tiletamine, 5.9 mg/ml and zolazepam, 5.9 mg/ml, (Zoletil vet, Virbac Laboratories, Carros, France), xylazine, 9.5 mg/ml, (Narcoxyl vet, Roche, Basel, Switzerland) and butorphanol, 0.23 mg/ml, (Torbugesic, Fort Dodge Laboratories, Fort Dodge, Iowa, USA). The initial dose of this mixture was 0.17 ml/100 g, and maintenance doses were 0.043 ml/100 g administered hourly. On completion of the experiments the animals were sacrificed using intracardial injections of penthobarbital (Haukeland Hospital Pharmacy, Bergen, Norway). During the experiments the body temperature was maintained at 37°C using a CMA/150 temperature controller (CMA/Microdialysis AB, Solna, Sweden). Infusion catheters for administering i.v. infusions and probes for performing MD were inserted through the right and left pectoral muscles into the external jugular veins through small skin incisions. Through a small midline laparotomy, placement of the MD probe in the extraperitoneal (XP) position in the flank was facilitated by tunneling outside the peritoneum with a blunt surgical probe under visual control. The MD probe was gently pushed in position beside the surgical probe which was then removed. MD probe or infusion catheter placement in the peritoneal cavity was accomplished through the abdominal wall and the muscle probe was placed in the left biceps femoris muscle through a separate skin incision. Except for the XP location, the probes and catheters were placed using an introducer consisting of a needle and split-tube.

### Stability of relative recovery during in vivo microdialysis

To evaluate the stability of RR in the compartments named above (vein, peritoneum, XP and muscle), three experiments were performed for each compartment. Because the equipment permitted only three MD probes per experiment, all compartments could not be examined simultaneously. After surgery the probes were flushed with R-A for 15 min at flow rate 10 μl/min, for the rest of the experiment the flow rate was 1 μl/min. After another 15 min with blank R-A, perfusion with 2 μM MMC in R-A was initiated and the probes were perfused for 90 min for equilibration of the system before start of sampling. MD was performed for seven hours with continuous sampling of 20-μl fractions, i.e. 21 replicates were collected from each probe. To avoid clotting on the vein probe, dalteparin 25 IE/ml (Fragmin, Pfizer, Limoges, France) was added to the perfusate in this probe. The concentration in the tissue being zero, RR of each sample collected was calculated using equation (3).

### Tissue distribution of mitomycin C during intravenous and intraperitoneal infusion

In this set of experiments, MD was performed to evaluate tissue distribution of MMC in rats during and after i.v. and i.p. bolus infusion. In the i.v. group the MD probes were placed in the vein, XP and peritoneum compartments and the infusion catheter in the left external jugular vein, while the i.p. group had probes in the vein, XP and muscle and an i.p. infusion catheter. For equilibration after surgery, the probes were perfused with R-A for 30 min and with 2 μM MMC in R-A for 90 min as described above. Individual calibration of the probes by the retrodialysis method was then performed in each experiment prior to the infusion of MMC. Four 10-μl samples were collected from each probe, and RR was calculated using equation (3) with C_out _defined as the mean MMC-concentration in the dialysates. After calibration, the remainder of the experiments were performed using blank R-A as perfusate, starting with a 30 min washout of the drug. MMC 2.5 mg/kg (750 μM in saline) was then infused over 30 min using an Asana syringe pump (Alaris medical systems, Basingstoke, UK). Dialysate sampling was started 6 min after infusion start, allowing time for emptying of the dead-volume of the probe. Continuous sampling was performed for four hours at flow rate 1 μl/min with sample volumes of 10 μl. The tissue concentration of MMC was calculated by reformulating equation (2), dividing the dialysate concentrations by RR determined by the probe calibration. Dalteparin was added to the perfusate in the vein probe throughout the experiments as described above. Blood samples for plasma analyses were taken from the lesser saphenous veins 35, 65 and 95 min after start of infusion, immediately centrifuged and plasma was stored at -70°C until analysis. To calculate the fraction of MMC absorbed from the abdominal cavity during the i.p. infusion experiments, the abdomen was opened at the end of sampling and the remaining fluid was collected for MMC content analysis.

### Calculations and statistical analysis

Pharmacokinetic parameters were determined by non-compartmental analysis using Kinetica ver 4.4.1 (Thermo Fisher Scientific Inc, Philadelphia, PA, USA). Statistical calculations were performed using SPSS software version 16.0 (SPSS GmBH, Chicago, Ill). Comparisons of area under the curve (AUC) in vein, peritoneum, XP, and muscle after i.v. and i.p. infusion were performed using the Mann-Whitney test, and p-values < 0.05 were considered significant.

## Results

### In vitro microdialysis in Ringer's - Acetate

**Figure 1:**
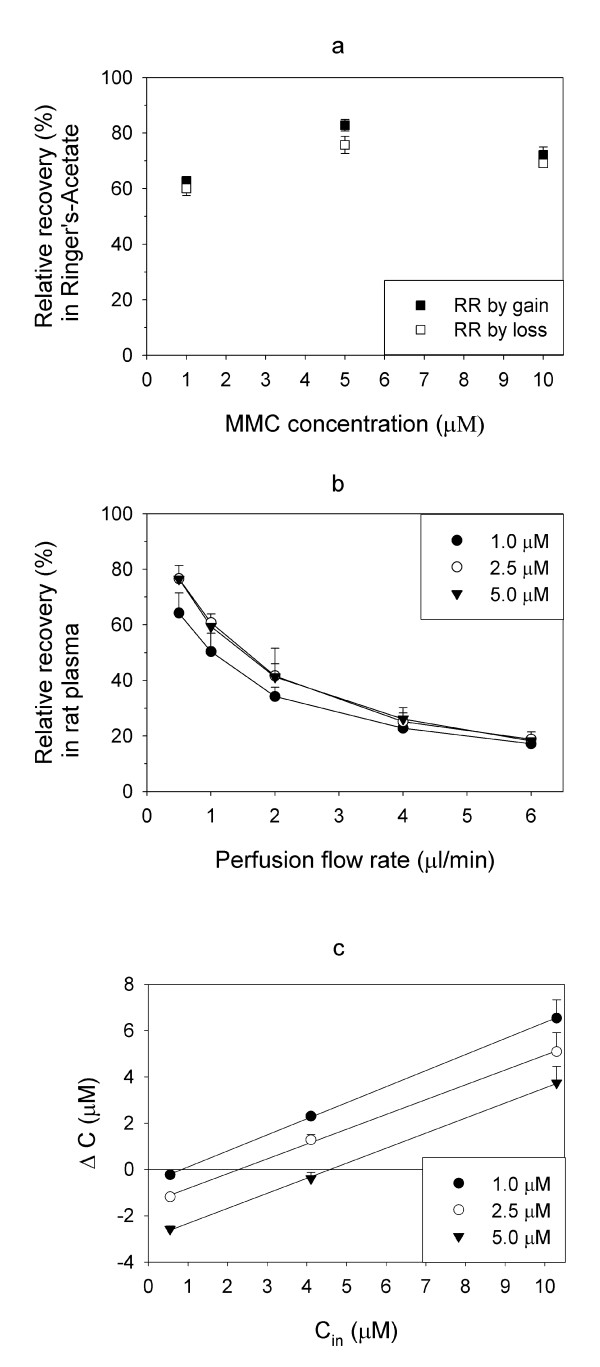
**In vitro microdialysis of mitomycin C (MMC)**. a. Comparison of in vitro relative recovery by gain (RR_gain_) and RR_loss_. In vitro RR_gain _and RR_loss _calculated from experiments with identical concentrations of MMC in Ringer's-Acetate for the individual probe. In each experimental setup 12 replicates were collected from each probe, with one probe for each concentration. The results are presented as mean RR (SD). b. The effect of perfusion flow rate on RR. The effect of perfusion flow rate on RR in three experiments with concentrations of MMC in rat plasma of 1.0, 2.5 and 5.0 μM, with one probe for each concentration. The flow was stepwise reduced from 6 to 0.5 μl/min. RR represent the relation between the dialyste concentration and total concentration of MMC. The results are presented as mean RR (pos.SD). c. In vitro zero-net flux (ZNF). ZNF was performed on MMC in rat plasma at concentrations of 1.0, 2.5 and 5.0 μM, with one probe for each concentration. The concentrations of MMC in perfusate were 0.5, 4 and 10 μM. Mean ΔC (=C_in_-C_out_) with pos SD calculated from three experiments. The interceptions of the regression lines on the x-axis are equal to the free concentrations of MMC in plasma, while the slopes of the regression lines represent RR for the probes.

For in vivo MD the probe is routinely calibrated by the retrodialysis method using equation (3) to calculate RR_loss_, whereas the actual tissue concentration after administration of the solute is calculated by equation (2), dividing the dialysate concentration by RR_gain_. Therefore, RR_gain _should be equal to RR_loss _for relevant concentrations of the solute [21,22]. Mean RR_gain _and RR_loss _for MMC using the presented experimental setup were 62.7% and 60.1% at 1 μM, 82.9% and 75.7% at 5 μM and 72.2% and 75.7% at 10 μM, respectively (figure 1a).

### In vitro microdialysis in plasma

During in vivo administration of a solute the tissue concentration will usually vary over time, in HIPEC the temperature is intentionally increased during the procedure, and finally, a flow rate that results in an acceptable RR with reasonable sample volumes must be chosen. To evaluate the influence of these parameters on RR, three series of in vitro experiments were performed with MMC in rat plasma. In the flow rate experiments mean RR increased from 18.1% at flow 6 μl/min to 72.5% at 0.5 μl/min. At 1 μl/min, RR was 50.4% at 1.0 μM, 60.8% at 2.5 μM and 59.4% at 5.0 μM, and this flow rate was used for the remainder of the experiments in the present work (figure 1b). To evaluate the effect of temperature on RR during MD, the medium temperature was varied in another set of experiments, and RR (SD) was 49.4% (5.0) at 37°C, 50.0% (9.7) at 40°C, 53.9% (10.1) at 42°C and 54.9% (10.9) at 44°C. The mean concentrations of free MMC in plasma calculated by the ZNF method were 0.9, 2.3 and 4.6 μM, while the total plasma concentrations in the same samples were 1.0, 2.5 and 5.0 μM. RR expressed by the equations of the linear regression lines were 69.2% at 1.0 μM, 64.0% at 2.5 μM and 65.0% at 5.0 μM, with correlation coefficients (r^2^) of all three regression lines above 0.999 (figure 1c). When comparing results from ZNF and flow rate experiments (1 μl/min), the calculated RR were relatively similar but generally slightly higher when the ZNF method was used.

### Stability of relative recovery during in vivo microdialysis

**Figure 2:**
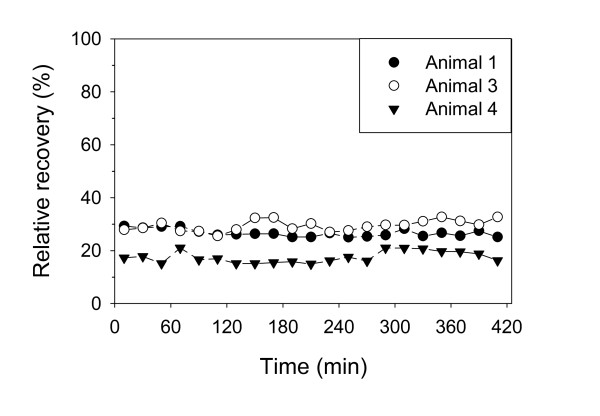
**In vivo calibration**. The curves depict relative recovery by loss of 21 replicates collected over seven hours in peritoneum from three different experiments.

In MD, long-term stability of RR is essential since an in vivo experiment includes both calibration of the probes and subsequent sampling of dialysates after administration of the solute in question. The results from in vivo calibration by the retrodialysis method, with mean RR (SD) calculated from 21 replicates from each probe, varied between 17.6% (2.2) in the peritoneum and 31.8% (4.4) in the vein (table 1). Figure 2 depicts RR results from three calibration experiments performed with the peritoneal MD probe, and illustrates both inter-experiment variation, but also that RR was relatively stable for each probe during seven hours of MD.

**Table 1:** Relative recovery by loss over seven hours in jugular vein, peritoneum, XP and biceps femoris muscle.

Animal number	Vein	Peritoneum	XP	Muscle
1	np	26.7 (1.4)	22.4 (3.9)	23.5 (1.6)
2	20.9 (3.4)	np	23.0 (2.3)	28.9 (3.1)
3	31.5 (2.9)	29.5 (2.1)	np	27.2 (1.9)
4	31.8 (4.4)	17.6 (2.2)	28.5 (1.1)	np

XP = extraperitoneal space, np = not performed.

Results are expressed as mean (SD) of 21 replicates per experiment and compartment.

### Tissue distribution of mitomycin C during intravenous and intraperitoneal infusion

**Figure 3:**
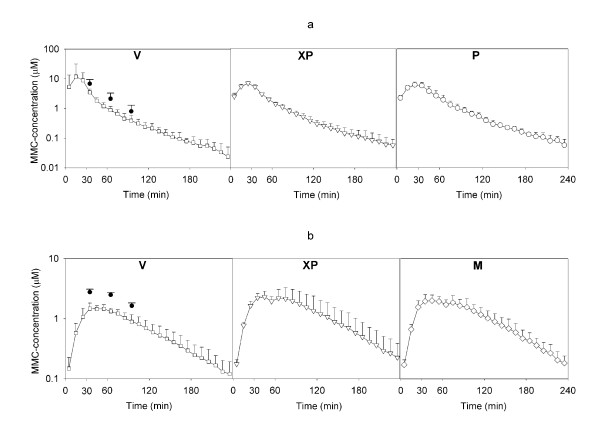
**a. Intravenous bolus infusion and b. Intraperitoneal bolus infusion**. a. Semilogaritmic concentration vs. time curves of mitomycin C (MMC) from microdialysis probes placed in vein (V), the extraperitoneal space (XP) and intraperitoneally (P) after 30 min i.v. infusion. Microdialysis sampling started six minutes after start of infusion to allow emptying of dead volume in microdialysis probes. The results are presented as mean (+ SD) from six experiments. The filled circles in the V diagram represent total serum MMC concentrations (+ SD). b. Semilogaritmic concentration vs. time curves of MMC from microdialysis probes placed in V, XP and hind leg muscle (M) after 30 min i.p. infusion. Microdialysis sampling started six minutes after start of infusion to allow emptying of dead volume in microdialysis probes. The results are presented as mean (+ SD) from five experiments. The filled circles in the V diagram represent total serum MMC concentrations (+ SD).

In the i.v. infusion experiments one of the peritoneal MD probes suffered a technical malfunction and the results from this probe have been omitted. In two of the animals in this group very high C_max _values were measured in the vein (51.2 and 15.6 μM), compared with C_max _values between 3.8-5.2 μM in the remaining four rats. However, immediately after terminating i.v. infusion the vein concentrations in all animals were almost the same and declined according to 1. order kinetic for the rest of the experiment. Median C_max _(μM) varied between 5.0 (range 3.8-51.2) in vein and 7.4 (range 3.7-7.9) in peritoneum. Median AUC (μM × min) varied between 202 (range 144-1038) in vein and 347 (range 219-425) in peritoneum with no significant difference between compartments. The half-life (min) of MMC in the various compartments was calculated from the concentration fall in the elimination phase of the i.v. infusion group, and varied between median 39.0 (range 26.2-50.4) in XP and 42.2 (range 25.4-51.2) in peritoneum (figure 3a). In the i.p. group the concentration curves were nearly similar in all compartments with a variation in median C_max _(μM) between 1.51 (range 1.29-1.94) in vein and 2.41 (range 1.88-4.07) in XP. Median AUC (μM × min) varied between 170 (range 105-229) in vein and 246 (range 165-534) in XP with no significant difference between the compartments (figure 3b). There was no significant difference in AUC between the i.v. and i.p. groups with respect to the vein and XP compartments. The pharmacologic parameters derived from the i.v. and i.p. infusion experiments are summarized in table 2.

**Table 2:** Pharmacokinetic parameters calculated after intravenous and intraperitoneal bolus infusion

	i.v. infusion			i.p. infusion		
	
	Vein	XP	Peritoneum	Vein	XP	Muscle
AUC (μM × min)	202 (144-1038)	311 (258-378)	347 (219-425)	170 (105-229)	246 (165-534)	234 (160-321)
t_½ _(min)	41.7 (34.2-54.6)	39.0 (26.2-50.4)	42.2 (25.4-51.2)			
C_max _(μM)	5.0 (3.8-51.2)	6.6 (5.8-8.3)	7.4 (3.7-7.9)	1.5 (1.3-1.9)	2.4 (1.9-4.1)	2.1 (1.4-2.9)
t_max _(min)	20-30	20-30	20-30	40-50	40-50	40-50

XP = extraperitoneal space, AUC = area under the curve, t_½ _= half-life, C_max _= maximal concentration, t_max _= time after start of infusion until maximal concentration with time intervals of 10 min.

The results are expressed as median (range) from six animals in the i.v. group and five animals in the i.p. group. For t_max_, the results represent the time interval with maximal concentration.

The ratio between the free concentration of MMC in blood determined by MD, and the total plasma concentration of MMC from blood samples collected at the same time, was 0.52 (SD 0.14), (n = 31). At the end of the experiments in the i.p. group the abdomen contained mean 3.0 ml (SD 0.3) with a mean MMC concentration of 1.9 μM (SD 1.3), i.e. 99.8% of the infused MMC was absorbed from the abdominal cavity during the experiments.

## Discussion

In the present work, the results from in vitro experiments have verified the feasibility of performing MD of MMC, and shown stability of RR over a relevant concentration range. By in vivo calibration using the retrodialysis method, stable RR over seven hours were accomplished in four tissue compartments in rats. In the subsequent use of MD in two groups of rats receiving i.v. and i.p. bolus infusions, a relatively fast and even distribution of MMC was observed, with no significant differences in AUC between compartments within each group, or when comparing compartments between the two groups.

The symmetry between RR_loss _and RR_gain _obtained by in vitro MD of MMC in R-A, indicated that MMC passed freely over the probe membrane did not bind to syringes and tubes. This is a prerequisite if measured dialysate concentrations are to immediately and consistently reflect the concentration of a solute in the medium outside the probe, and for the validity of calibration by the retrodialysis method [21]. The observed variation in RR between the three probes used was not concentration dependent, and it was therefore interpreted to be related to a certain individual variation in membrane permeability between the probes, underscoring the necessity for individual probe calibration [19].

Both the flow rate and ZNF experiments allowed for evaluation of stability of RR over the concentration range 1.0-5.0 μM, which is a relevant interval based on measured plasma-concentrations in patients during HIPEC [6]. RR obtained by the ZNF method was almost identical in the three concentrations studied, as illustrated by the parallel slopes of the regression lines (figure 1c). In the flow rate experiments, mean RR at 2.5 and 5.0 μM were also very similar for all flow rates, while the slightly divergent result at 1.0 μM was caused by very low RR obtained in one of the three experiments. The apparent discrepancy between RR determined by ZNF and flow rate experiments at 1 μl/min should be interpreted in view of MMC plasma protein binding previously shown to be approximately 10% [31]. RR determined by the ZNF method was obtained solely by MD, i.e. reflecting the free fractions of MMC, while in the flow rate experiments, RR was calculated by equation (2), where the dialysate concentrations were divided by total plasma concentrations (free and protein-bound). Equivalently, the overall difference between the free MMC concentrations calculated by ZNF and total plasma concentrations may also be explained by protein binding. In summary, acceptable stability of RR in the concentration range of 1.0-5.0 μM was demonstrated by the in vitro experiments on MMC in plasma.

In vivo RR will partly be determined by factors likely to vary between otherwise identical experimental setups, such as the diffusion and clearance rate of the solute in the probe membrane vicinity which will be influenced by circulatory conditions of the tissue [20]. A certain difference in membrane permeability between presumed identical probes (i.e. the same product) as described by others and observed in this work, will also contribute to RR variation [19]. The variation between RR of probes inserted in the same compartment in this work illustrates the importance of individual probe calibration and hence the need of a straightforward calibration method. Because the ZNF method is time-consuming and requires steady-state tissue concentration of the solute it is considered an inconvenient method for in vivo calibration, whereas the retrodialysis method prior to administration of the drug in question is fast and reliable and the most commonly used in vivo calibration method [21]. A special precaution had to be taken to achieve acceptable long-term (~7 h) stability of RR in the vein probe, i.e. low molecular heparin was added to the perfusate throughout the experiment to prevent a gradual fall in RR which was observed in initial experiments and probably was caused by clotting on the probe membrane.

After i.v. bolus infusion, C_max _values were higher and reached more rapidly than in the i.p. infusion group. This was expected, since in the latter group the abdominal cavity served as a functional reservoir wherefrom MMC was absorbed throughout the experiments, entailing a slower and more prolonged systemic exposure of the drug compared with i.v. infusion. Since almost all MMC infused i.p. was absorbed to the systemic circulation, both i.v. and i.p. infusion represented de facto systemic administration modalities. No significant differences in AUC in the same compartments between the two groups indicate equal bioavailability is achieved with i.v. and i.p. infusion strategies.

With the exception of the vein compartment in the previously mentioned two animals during ongoing i.v. infusion, the similarity between concentration curves of the three compartments within each experimental setup reflects a fast end even distribution of MMC. In analogy with conclusions drawn by other groups who have performed MD on other water soluble drugs, this indicates that measured concentrations in easily accessible muscle tissues are representative of systemic MMC concentration [32]. Although tissue distribution was fast and even in the examined tissues, it should be noted that local drug efficacy and toxicity might also depend on enzymatic activation rates in specific tissues [33]. The half-life could not be calculated after i.p. infusion, since 1. order kinetic was not achieved in this group because of continuous absorption from the abdominal cavity. The calculated MMC half-life of 42 min following i.v. administration is somewhat higher than reported by others (25 min), but the results may not be directly comparable since different strains of rats were used [34]. In two series of patients treated for peritoneal carcinomatosis with MMC administered in the form of HIPEC, mean plasma half-lives of 76 and 84 min were found, in agreement with the general pharmacological observation of slower drug metabolism in larger organisms [6,14].

## Conclusions

Results from the i.v. and i.p. infusion experiments confirmed that MD can be used to monitor MMC tissue concentrations in vivo, also illustrated by the reproducible relationship between concentrations calculated by intravascular MD and plasma concentrations in blood samples. The moderate increase in RR with rising temperature has to be taken into consideration, and may imply extra attention when planning and interpreting experiments involving differences in temperature between probe calibration and subsequent dialysate sampling. With these precautions in mind, the use of MD for MMC monitoring can probably be extended to be performed experimentally in rats during HIPEC and eventually in humans.

## Competing interests

The authors declare that they have no competing interests.

## Authors' contributions

OS participated in study design, data collection and interpretation and preparation of the manuscript. AA participated in study design, drug analysis, data interpretation and preparation of the manuscript. HO participated in study design and data interpretation. AK participated in data collection and interpretation. POE participated in study design and data collection. KEG participated in study design and data interpretation. KF participated in study design, data interpretation and preparation of the manuscript. All authors read and approved the final manuscript.

## Pre-publication history

The pre-publication history for this paper can be accessed here:

http://www.biomedcentral.com/1471-2407/10/469/prepub
